# Clinical characteristics, risk factors and outcome of critically ill immunocompromised patients with bloodstream infections and sepsis

**DOI:** 10.1371/journal.pone.0332807

**Published:** 2025-09-24

**Authors:** Nirusdee Vonineng, Yuda Sutherasan, Jackrapong Bruminhent

**Affiliations:** 1 Department of Medicine, Faculty of Medicine Ramathibodi Hospital, Mahidol University, Bangkok, Thailand; 2 Division of pulmonary and Pulmonary Critical care Medicine, Department of Medicine, Faculty of Medicine Ramathibodi Hospital, Mahidol University, Bangkok, Thailand; 3 Division of Infectious Diseases, Department of Medicine, Faculty of Medicine Ramathibodi Hospital, Mahidol University, Bangkok, Thailand; Children's National Hospital, George Washington University, UNITED STATES OF AMERICA

## Abstract

**Background:**

Immunocompromised patients with sepsis face higher mortality than immunocompetent individuals. However, data on bloodstream infections (BSIs) and sepsis among critically ill immunocompromised (CII) patients remain limited. We aimed to describe the epidemiology, outcomes, and mortality risk factors of BSIs in this population.

**Methods:**

We conducted a retrospective cohort study of CII patients admitted to the medical ICU between January 2022 and December 2023 with suspected sepsis or septic shock. Patients with BSIs confirmed by positive blood cultures were identified. Propensity score matching (1:1) without replacement was used to create comparable groups for Cox regression analysis of 30-day all-cause mortality.

**Results:**

Among 211 CII patients (mean age (SD) 61 (16) years, 57% male), 85 (40.3%) had BSIs. The median SOFA and APACHE II scores were 7 (IQR 4–11) and 16 (IQR 14–20), respectively. Immunosuppression was due to hematologic malignancy (37.4%), solid tumors (27.0%), autoimmune diseases (19.0%), solid organ transplantation (5.7%), and other causes (10.4%). Gram-negative rods predominated (65.9%), notably *P. aeruginosa* (17%), *E. coli* (17%), and *K. pneumoniae* (14%). The overall 30-day mortality rate was 48.8%. In the matched cohort (n = 170), higher SOFA scores [HR 1.12; 95% CI, 1.04–1.20; p = 0.003] and lactate >4 mmol/L [HR 1.91; 95% CI, 1.06–3.42; p = 0.031] were associated with increased mortality. Underlying COPD/asthma was associated with lower mortality [HR 0.20; 95% CI, 0.06–0.66; p = 0.009].

**Conclusion:**

BSIs are frequent in CII patients and linked to high mortality. Severity of illness and hyperlactatemia predict poor outcomes, while preexisting pulmonary disease may offer a survival benefit.

## Introduction

Sepsis remains among the most common complications from infectious disease worldwide. It is a life-threatening condition resulting from a dysregulated host response to infection [1]. Sepsis can cause significant morbidity and mortality among patients admitted to an intensive care unit.

An immunocompromised patient with sepsis has greater odds of in-hospital mortality compared to immunocompetent patients [2,3]. Individuals with cancer exhibited a higher odds ratio of mortality at 28 days compared to the other immunocompromised groups [3]. Early and adequate antimicrobial therapy is crucial for improving patient outcomes, particularly in those meeting criteria for sepsis or septic shock. It should be guided by established guidelines and direct examination of available samples [4].

Few studies have focused on bloodstream infections (BSIs) and sepsis in critically ill immunocompromised (CII) patients, especially in the Thai population. Additionally, predictors to guide clinicians for prognosis among CII patients, with and without BSI, are lacking and mandatory to explore.

The primary objective of this study is to investigate the epidemiology of pathogens causing BSI and sepsis in CII patients. Additionally, secondary objectives encompass assessing the outcomes of BSI, evaluating the characteristics of sepsis, and investigating risk factors for both BSI and mortality in this specific patient population.

## Methods

A retrospective cohort study was undertaken at the Medical Intensive Care Unit of Ramathibodi Hospital, Bangkok, Thailand, spanning from January 2022 to December 2023. Patient data were accessed between February 20, 2023, and February 15, 2024. The study focused on participants characterized by immunocompromised status, suspected sepsis, and septic shock, who subsequently developed BSI confirmed by positive blood cultures.

Our inclusion criteria were: immunocompromised patients with any of the following conditions—primary immunodeficiency, active malignancy or malignancy diagnosed within one year and receiving chemotherapy in the past three months, HIV infection with CD4 count < 200 cells/mm³, solid organ or hematopoietic stem cell transplantation, neutropenia (ANC < 500 cells/mm³), treatment with biologic immune modulators, or use of DMARDs or other immunosuppressive drugs—who had a Ramathibodi Early Warning Score (REWS) ≥ 2 with suspected infection, were admitted to the medical ICU, and were aged 15 years or older.

On the other hand, patients with incomplete medical records, those referred from another hospital, individuals requesting a Do Not Resuscitate (DNR) order on the first day of admission, and those lost to follow-up within 90 days after admission were excluded from the study.

The cumulative 30-, 60-, and 90-day mortality rates were estimated using the Kaplan-Meier methodology. Propensity score matching at a 1:1 ratio without replacement was performed to create comparable groups. These matched groups were then utilized in Cox proportional hazards modeling to estimate the risk of all-cause mortality within 30 days.

This trial was received approval from the Institutional Review Boards at Faculty of Medicine Ramathibodi Hospital, Mahidol University, Bangkok, Thailand. (COA. MURA2023/157). Written informed consent was exempted by the Ethics Committee due to the retrospective nature of the study and the assurance of participant confidentiality in accordance with ethical policies.

### Statistical analyses

The data were characterized using mean and standard deviation (SD) or and interquartile range (IQR) median for continuous variables, and percentages for categorical variables. Baseline categorical variables were compared between groups using the Chi-square or Fisher exact test as appropriate, while baseline continuous variables were compared using the Student T-test or Wilcoxon rank-sum test. Cox proportional hazards modeling was employed to estimate the unadjusted hazard ratio (HR) and its associated 95% confidence interval (CI) for factors linked to the time to 30-day mortality. Propensity scores were computed through a multivariable logistic regression model, encompassing all covariates, and score matching was conducted with a caliper of 0.2 to generate the propensity score. Standardized mean biases were assessed to ensure balance post-propensity score matching between groups. All statistical analyses were carried out using STATA version 18.0 (StataCorp®, TX), and statistical significance was set at a P-value < 0.05 (2-sided).

## Results

### Study population

**Fig 1:**
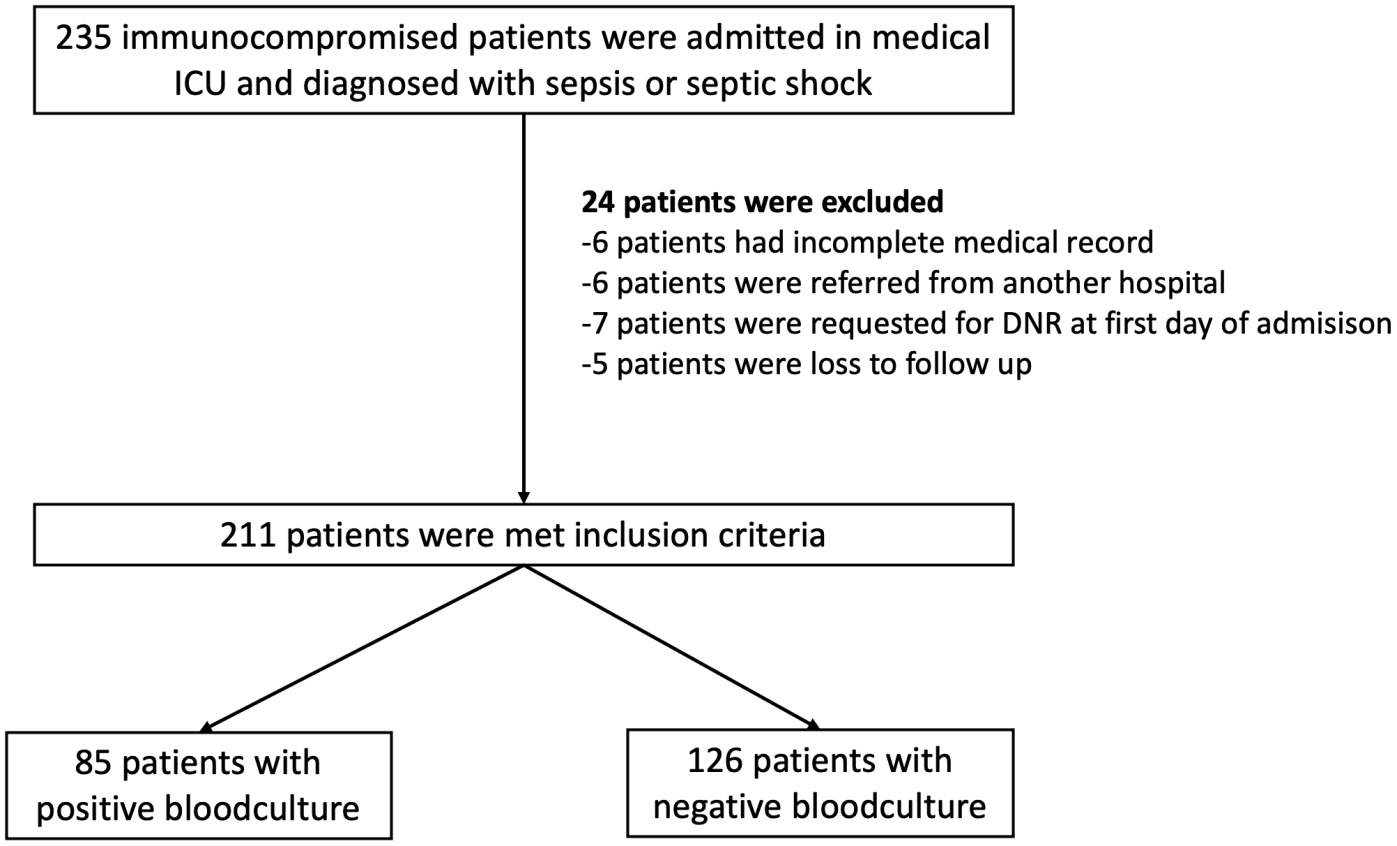
Study flow chart. Abbreviations ICU: Intensive Care Unit, DNR: Do Not Resuscitation.

A total of 235 immunocompromised patients were admitted to the medical ICU and diagnosed with sepsis or septic shock. Twenty-four patients were excluded: 6 patients had incomplete medical records, 6 patients were referred from another hospital, 7 patients requested a DNR (Do Not Resuscitate) on the first day of admission, and 5 patients were lost to follow-up, resulting in 211 patients being included (Fig 1) (Supporting File 1) The mean age (SD) was 61 (16) years, and 57% were male, the immunodeficiency profile included hematologic malignancy (37.4%), solid organ malignancy (27.0%), autoimmune disease (19.0%), solid organ transplantation (5.7%), others (10.4%).42 patients (19.9%) had diabetes mellitus, 25 patients (11.8%) had a cardiac underlying disease and 18 (8.5%) had a COPD or asthma. Furthermore, 101 patients (47.9%) were diagnosed with pulmonary infections (Table 1).

**Table 1:** Baseline characteristic of critically-ill immunocompromised patients with bloodstream infection.

Characteristic	n (%)
Age (years), mean (SD)	60.6 (16.1)
Gender, n (%)	
Male	119 (56.4)
Female	92 (43.6)
Underlying disease, n (%)	
• DM	42 (19.9)
• COPD/Asthma	18 (8.5)
• Cirrhosis	7 (3.3)
• Heart disease	25 (11.8)
• CKD stage ≥ 3	16 (7.6)
• Hyperthyroid/Hypothyroid	7(3.3)
• Stroke	8(3.8)
- Immunodeficiency profile, n (%)	
• HIV infection	10(4.7)
• Solid organ transplantation	12(5.7)
• Hematopoietic stem cell transplantation	10(4.7)
• Autoimmune disease	40 (19.0)
• Hematologic malignancy	79(37.4)
• Solid organ malignancy	57(27.0)
• Other immune deficiency	3(1.4)
Source of infection, n (%)	
• Pulmonary	101(47.9)
• Genitourinary	23(10.9)
• Gastrointestinal	37(17.5)
• Mucocutaneous	5(2.4)
• Central Nervous System	0(0)
• Unknown	45(21.3)

**Abbreviations** CKD: Chronic Kidney Disease, COPD: Chronic Obstructive Pulmonary Disease, DM: Diabetes Mellitus, HIV: Human Immunodeficiency Virus.

### Bloodstream infections

**Fig 2:**
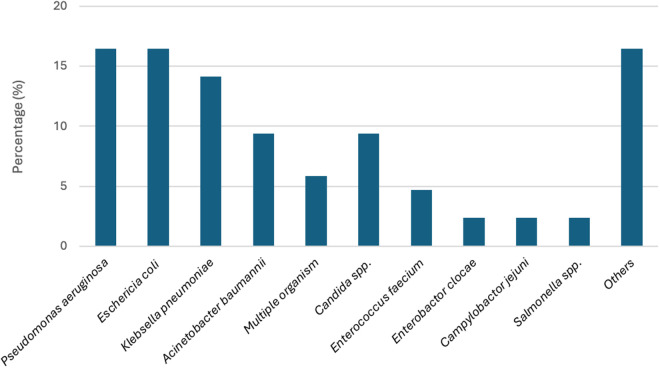
Pathogens recovered from blood culture in critically-ill immunocompromised patients with bloodstream infection. Others included: *Fusarium* spp. (n = 1), *Bacillus* spp. (n = 1), *Cryptococcus neoformans* (n = 1), *Lomentospora prolificans* (n = 1), *Lysinibacillus* spp. (n = 1), *Ralstonia mannitolilytica* (n = 1), *Roseomonas mucosa* (n = 1), *Streptococcus agalactiae* (n = 1), *Staphylococcus aureus* (n = 1), *Stenotrophomonas maltophilia* (n = 1), *Corynebacterium* spp. (n = 1), and *Achromobacter xylosoxidans* (n = 1).

There were 85 cases (40.3%) with positive blood cultures, predominately showing gram negative-rod bacteria (65.9%), fungus (16.5%) and gram-positive bacteria (11.7%) (Fig 2). The predominant pathogens included *Pseudomonas aeruginosa* (17%), *Escherichia coli* (17%), and *Klebsiella pneumoniae* (14%). Other pathogens identified were *Acinetobacter baumannii* (10%), multiple pathogens (9%), *Candida* spp. (9%), *Enterococcus faecium* (5%), *Enterobacter cloacae* (2%), *Campylobacter jejuni* (2%), and *Salmonella* spp. (2%). The remaining 13% of isolates included: *Fusarium* spp. (n = 1), *Bacillus* spp. (n = 1), *Cryptococcus neoformans* (n = 1), *Lomentospora prolificans* (n = 1), *Lysinibacillus* spp. (n = 1), *Ralstonia mannitolilytica* (n = 1), *Roseomonas mucosa* (n = 1), *Streptococcus agalactiae* (n = 1), *Staphylococcus aureus* (n = 1), *Stenotrophomonas maltophilia* (n = 1), *Corynebacterium* spp. (n = 1), and *Achromobacter xylosoxidans* (n = 1).

### Mortality

**Fig 3:**
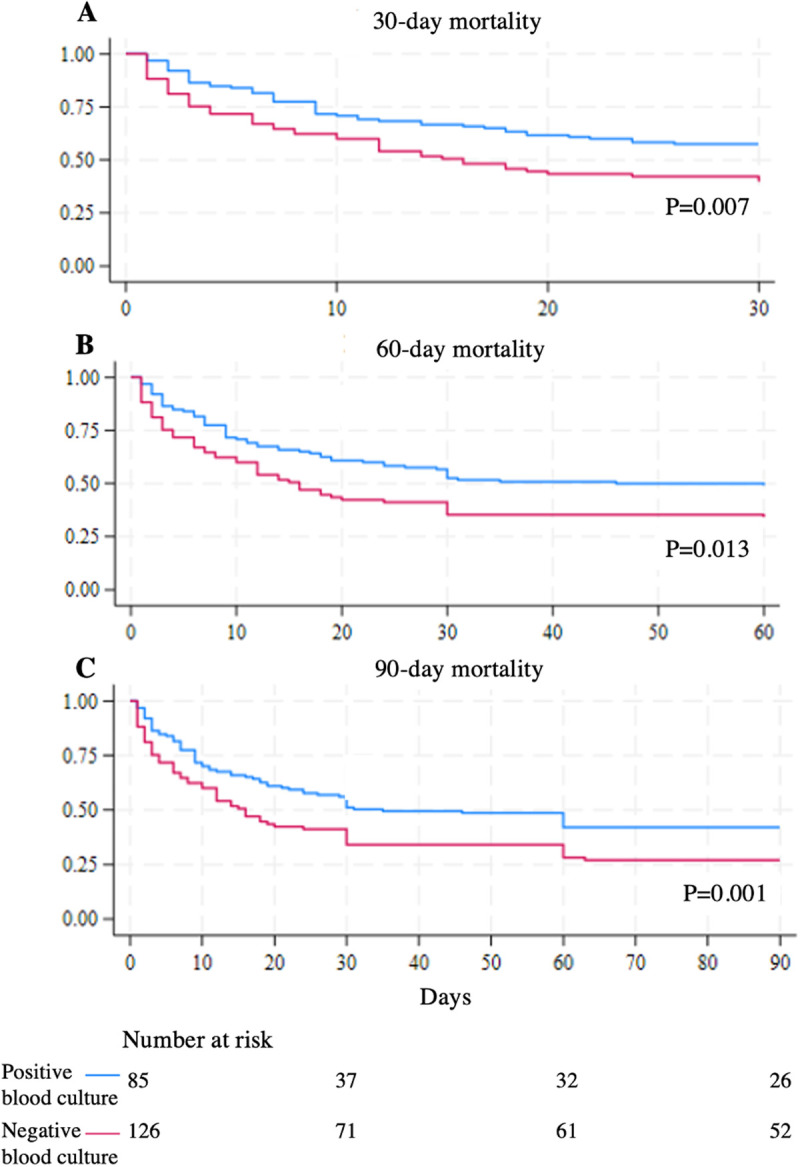
Kaplan–Meier curves depicting (A) 30-, (B) 60-, and (C) 90-day mortality among critically ill immunocompromised patients stratified by the presence or absence of bloodstream infection. Survival probabilities are plotted over time, with corresponding numbers at risk shown for each time point. Overall, 103, 118, and 133 patients died within 30, 60, and 90 days, respectively.

The Kaplan-Meier curve between CII patients with positive and negative blood culture were compared, depicting 30-, 60-, and 90-day mortality, revealed that patients with positive blood culture had significantly higher mortality rates at 30-, 60-, and 90-days, with p-values of 0.007, 0.013, and 0.001, respectively (Fig 3).

In the entire cohort, age, gender, solid organ malignancy, pulmonary and gastrointestinal infectious sources were found to be significantly different between those with and without BSI. However, in a propensity score–matched cohort, there were 85 well-balanced matches paired to positive and negative blood culture groups, respectively. There were no statistically significant differences in baseline characteristics between the two groups (Table 2)

**Table 2:** Baseline characteristics of whole cohort and propensity score–matched cohort between critically-ill immunocompromised patients with and without bloodstream infection.

Characteristic	Whole cohort (n= 211)			Propensity score–matched cohort (n= 170)		
	Positive blood culture	Negative blood culture	P-value	Positive blood culture	Negative blood culture	P-value
	n=85	n=126		n=85	n=85	
Age, years, mean (SD)	57.7 (15.7)	62.6 (16.2)	0.032	59.5 (1.9)	60.5 (1.5)	0. 255
Male gender, n (%)	41 (48.2)	78 (61.9)	0.050	47 (55.3)	47 (55.3)	1.000
Underlying disease, n (%)						
• DM	17 (20.0)	25 (19.8)	0.980	20 (23.1)	19 (22.7)	0.855
• COPD/Asthma	5 (5.9)	13 (10.3)	0.260	4 (5.2)	7 (7.9)	0.350
• Cirrhosis	2 (2.4)	5 (4.0)	0.520	4 (4.2)	3 (3.4)	0.350
• Heart disease	9 (10.6)	16 (12.7)	0.640	9 (10.8)	10 (11.6)	0.808
• CKD stage ≥ 3	7 (8.2)	9 (7.1)	0.770	6 (7.3)	6 (7.3)	1.000
• Hyperthyroid/hypothyroid	3 (3.5)	4 (3.2)	0.890	3 (3.1)	2 (2.9)	1.000
• Stroke	2 (2.4)	6 (4.8)	0.370	3 (3.6)	3 (3.6)	1.000
Immunodeficiency profile, n (%)						
• HIV infection	3 (3.5)	7 (5.6)	0.500	2 (1.9)	4 (3.8)	0.683
• SOT	4 (4.7)	8 (6.3)	0.610	4 (3.5)	5 (4.9)	1.000
• HSCT	4 (4.7)	6 (4.8)	0.990	5 (4.9)	5 (4.7)	1.000
• Autoimmune disease	20 (23.5)	20 (15.9)	0.160	26 (26.4)	20 (20.0)	0.313
• Hematologic malignancy	36 (42.4)	43 (34.1)	0.230	44 (44.0)	38 (38.0)	0.388
• Solid organ malignancy	16 (18.8)	41 (32.5)	0.028	35 (35.0)	45 (44.6)	0.149
Source of infection, n (%)						
• Pulmonary tract	27 (31.8)	74 (58.7)	<0.001	23 (23.2)	29 (29.0)	0.333
• Genitourinary tract	11 (12.9)	12 (9.5)	0.430	14 (14.4)	10 (10.0)	0.384
• Gastrointestinal tract	24 (28.2)	13 (10.3)	<0.001	40 (40.0)	49 (48.5)	0.200
• Mucocutaneous system	3 (3.5)	2 (1.6)	0.360	4 (4.4)	5 (4.8)	0.733
• Unknown	20 (23.5)	25 (19.8)	0.660	NA	NA	NA

**Abbreviations** CKD: chronic kidney disease, CNS: central nervous system, COPD: chronic obstructive pulmonary disease, DM: diabetes mellitus, HIV: human immunodeficiency virus, HSCT: hematopoietic stem cell transplantation, SOT: solid organ transplantation.

A total 103 (48.82%) patients died within 30 days. In the whole cohort, an initial lactate level above 4 mmol/L and a lactate level above 4 mmol/L at 6 hours post-resuscitation were found in the greater proportion of those with 30-day mortality, as well as higher scores across all sepsis assessment tools, including REWS, National Early Warning Score (NEWS), Sequential Organ Failure Assessment (SOFA), Quick Sequential Organ Failure Assessment (qSOFA), Acute Physiologic Assessment and Chronic Health Evaluation II (APACHE II), and Pitt Bacteremia Score. Furthermore, a greater proportion of patient with 30-day mortality was also found to have fungemia compared to those who survived (Table 3).

**Table 3:** Characteristics of critically-ill immunocompromised patients with and without 30-days mortality.

Factors	30-days mortality		P-value
	Death	Survived	
	n=103	n=108	
Age, years, mean (SD)	59.4 (17.0)	61.8 (15.2)	0.270
Male gender, n (%)	55 (46.2)	64 (53.8)	0.390
Underlying disease, n (%)			
• DM	21 (50.0)	21 (50.0)	0.860
• COPD/Asthma	4 (22.2)	14 (77.8)	0.018
• Cirrhosis	5 (71.4)	2 (28.6)	0.220
• Heart disease	14 (56.0)	11 (44.0)	0.440
• CKD stage ≥ 3	10 (62.5)	6 (37.5)	0.250
• Hypothyroid/hyperthyroid	5 (71.4)	2 (28.6)	0.220
• Stroke	1 (12.5)	7 (87.5)	0.036
Immunodeficiency profile, n (%)			
• HIV infection	4 (40.0)	6 (60.0)	0.570
• SOT	9 (75.0)	3 (25.0)	0.062
• HSCT	5 (50.0)	5 (50.0)	0.940
• Autoimmune disease	22 (55.0)	18 (45.0)	0.380
• Hematologic malignancy	36 (45.6)	43 (54.4)	0.470
• Solid organ malignancy	27 (47.4)	30 (52.6)	0.800
• Other immune deficiency	0 (0.0)	3 (100.0)	0.088
Source of infection, n (%)			
• Pulmonary tract	54 (53.5)	47 (46.5)	0.200
• Genitourinary tract	9 (39.1)	14 (60.9)	0.320
• Gastrointestinal tract	19 (51.4)	18 (48.6)	0.730
• Mucocutaneous system	2 (40.0)	3 (60.0)	0.690
• Central nervous system	0 (0.0)	0 (0.0)	N/A
• Unknown	20 (44.4)	25 (55.5)	0.400
Initial lactate >4 mmol/L, n (%)	39 (62.9)	23 (37.1)	0.008
Lactate at 6 hours >4 mmol/L, n (%)	35 (70.0)	15 (30.0)	0.001
Sepsis score, mean (SD)			
• REWS	8.2 (2.7)	6.8 (2.2)	<0.001
• NEWS	12.1 (2.9)	10.9 (2.8)	0.002
• APACHE	19.2 (6.3)	15.4 (4.6)	<0.001
• SOFA, median (IQR)	9 (6, 13)	6 (3, 8)	<0.001
• qSOFA	2.3 (0.7)	1.9 (0.7)	<0.001
• PTS, median (IQR)	5 (3, 6)	3 (2, 4)	<0.001
Receiving antibiotic ≤ 1 hour, n (%)	83 (45.4)	100 (54.6)	0.010
Pathogens, n (%)			
• Gram-negative bacteria	31 (55.4)	25 (44.6)	0.253
• Gram-positive bacteria	4 (40.0)	6 (60.0)	0.749
• Fungus	12 (85.7)	2 (14.3)	0.004
• Multiple organisms	4 (80.0)	1 (20.0)	0.158

**Abbreviations** CKD: chronic kidney disease, CNS: central nervous system, COPD: chronic obstructive pulmonary disease, DM: diabetes mellitus, HIV: human immunodeficiency virus, HSCT: Hematopoietic Stem Cell Transplantation, SOT: Solid Organ Transplantation.

In multivariate analyses of the whole cohort, higher SOFA scores were identified as risk factors associated with 30-day mortality [HR 1.11 (1.04, 1.18) P = 0.002]. Conversely, patients with COPD or asthma exhibited a protective factor [HR 0.30 (0.11, 0.85) P = 0.023] (Table 4).

**Table 4:** Factors associated with 30-days mortality in whole cohort by cox proportional hazards model.

Factors	Univariate analysis		Multivariate analysis	
	HR (95% CI)	P-value	HR (95% CI)	P-value
Positive blood culture	1.67 (1.14, 2.46)	0.009	0.89 (0.56, 1.41)	0.608
Age, years	0.99 (0.98, 1.01)	0.329	–	–
Male gender	1.18 (0.80, 1.74)	0.399		
Diabetes mellitus	0.98 (0.61, 1.58)	0.934		
COPD/Asthma	0.35 (0.13, 0.94)	0.038	0.30 (0.11, 0.85)	0.023
Cirrhosis	1.67 (0.68, 4.09)	0.266		
Heart disease	1.43 (0.81, 2.51)	0.216		
CKD stage ≥ 3	1.48 (0.77, 2.84)	0.239		
Hypothyroid/hyperthyroid	1.68 (0.68, 4.13)	0.257		
Stroke	0.19 (0.03, 1.37)	0.099		
SOT	1.73 (0.87, 3.43)	0.117		
HSCT	1.04 (0.42, 255)	0.933		
Autoimmune disease	1.26 (0.79, 2.02)	0.337		
Hematologic malignancy	0.88 (0.59, 1.33)	0.551		
Solid organ malignancy	0.94 (0.61, 1.46)	0.798		
Pulmonary tract	1.17 (0.80, 1.73)	0.416		
Genitourinary tract	0.80 (0.41, 1.59)	0.531		
Gastrointestinal tract	1.19 (0.72, 1.96)	0.488		
Mucocutaneous system	0.72 (0.18, 2.93)	0.649		
REWS	1.21 (1.12, 1.30)	<0.001	1.07 (0.94, 1.21)	0.336
NEWS	1.14 (1.07, 1.22)	<0.001	1.04 (0.92, 1.19)	0.496
APACHEII	1.10 (1.01, 1.13)	<0.001	1.01 (0.96, 1.05)	0.824
SOFA	1.16 (1.12, 1.21)	<0.001	1.11 (1.04, 1.18)	0.002
qSOFA	2.03 (1.52, 1.71)	<0.001	1.13 (0.71, 1.78)	0.606
PTS	1.29 (1.18, 1.40)	<0.001	0.97 (0.82, 1.15)	0.742
Initial lactate > 4 mmol/L	1.96 (1.32, 2.93)	0.001	1.29 (0.75, 2.23)	0.365
Lactate at 6 hours >4 mmol/L	2.55 (1.69, 3.84)	<0.001	1.51 (0.83, 2.76)	0.178
Receiving antibiotic ≤ 1 hour	0.58 (0.36, 0.95)	0.029	0.65 (0.38, 1.12)	0.122
Gram-negative bacteria	1.25 (0.81, 1.91)	0.301		
Gram-positive bacteria	0.59 (0.19, 1.87)	0.373		
Fungus	3.04 (1.62, 5.70)	0.001	1.70 (0.83, 3.51)	0.148
Multiple organisms	1.83 (0.67, 4.98)	0.235		

**Abbreviations** APACHE II: Acute Physiologic Assessment and Chronic Health Evaluation II, CKD: chronic kidney disease, CNS: central nervous system, COPD: chronic obstructive pulmonary disease, DM: diabetes mellitus, HIV: human immunodeficiency virus, HSCT: allogeneic stem cell tranplant, NEWS: National Early Warning score, PTS: Pitt Bacteremia Score, qSOFA: Quick Sequential Organ Failure Assessment, REWS: Ramathibodi Early Warning score, SOFA: Sequential Organ Failure Assessment, SOT: Solid Organ Transplantation.

Finally, in a propensity score–matched cohort, factors associated with 30-day mortality using a multivariate analyses mortality included a higher SOFA score and lactate level above 4 mmol/L, showing a trend towards increased risk with [HR 1.12 (95%CI, 1.04–1.20, P = 0.003] and [HR 1.91 (95%CI, 1.06–3.42, P = 0.031)], respectively. Additionally, underlying COPD/asthma appeared to be a protective factor [HR 0.20(95% CI, 0.06–0.66, P = 0.009)] (Table 5).

**Table 5:** Factors associated with 30-days mortality in propensity score–matched cohort by cox proportional hazards model.

Factors	Univariate analysis		Multivariate analysis	
	HR (95% CI)	P-value	HR (95% CI)	P-value
Positive blood culture	1.69 (1.12, 2.54)	0.012	1.03 (0.66, 1.61)	0.909
Underlying disease, COPD/Asthma	0.27 (0.10, 0.75)	0.012	0.20 (0.06, 0.67)	0.009
Initial lactate >4 mmol/L	2.00 (1.30, 3.07)	0.002	1.40 (0.83, 2.36)	0.202
Lactate at 6 hours >4 mmol/L	2.79 (1.76, 4.44)	<0.001	1.98 (1.11, 3.52)	0.020
Sepsis score				
• REWS	1.20 (1.09, 1.31)	<0.001	1.04 (0.90, 1.20)	0.625
• NEWS	1.15 (1.07, 1.25)	<0.001	1.11 (0.97, 1.27)	0.129
• APACHEII	1.08 (1.03, 1.13)	0.001	0.98 (0.94, 1.03)	0.483
• SOFA	1.14 (1.09, 1.20)	<0.001	1.11 (1.04, 1.20)	0.003
• qSOFA	2.02 (1.44, 2.84)	<0.001	1.08 (0.66, 1.78)	0.751
• PTS	1.24 (1.13, 1.36)	<0.001	0.96 (0.77, 1.20)	0.721
Receiving antibiotic ≤ 1 hour	0.58 (0.36, 0.91)	0.019	0.73 (0.41, 1.29)	0.278
Fungus	2.90 (1.47, 5.71)	0.002	1.74 (0.85, 3.53)	0.127

**Abbreviations** APACHE II: Acute Physiologic Assessment and Chronic Health Evaluation II, ATB: antibiotics, COPD: chronic obstructive pulmonary disease, NEWS: National Early Warning Score, PTS: Pitt Bacteremia Score, qSOFA: Quick Sequential Organ Failure Assessment, REWS: Ramathibodi Early Warning score, SOFA: Sequential Organ Failure Assessment.

## Discussion

Our findings reveal that gram-negative bacteria constitute the predominant organisms responsible for BSI in patients with immunocompromised conditions, comprising two third of cases. Specifically, our focus was on *P. aeruginosa*, *E. coli*, and *K. pneumoniae*. This pattern is likely due to the high prevalence of hospital-associated infections among the majority of patients. Additionally, pulmonary and gastrointestinal sources emerged as the primary origins of infection in these cases. Most of these BSIs caused by opportunistic pathogens are usually accompanied by oral mucositis and ulceration during chemotherapy, which may increase the risk of organism dissemination from the oral cavity to the bloodstream [5].

A previous post hoc analysis of a prospective, multicenter, multinational cohort revealed that gram-negative rod bacteria accounted for approximately 50% of bloodstream infections in immunocompromised patients with acute respiratory failure, with the pulmonary system being the primary source. However, gram-negative rod bacteremia was not found to be directly associated with increased mortality in this cohort [6]. In contrast, a comprehensive review focusing on septic shock in immunocompromised cancer patients identified gram-negative bacteremia, the nature and timing of initial treatment responses, and the degree of immunosuppression as key factors contributing to an elevated risk of progression to septic shock [7]. Additionally, multidrug-resistant Gram-negative rod bacteria are a well-recognized cause of bacteremia among kidney transplant recipients, particularly those infected with strains producing extended-spectrum beta-lactamase enzymes [8]. Moreover, we identified emerging pathogens such as *C. jejuni*, which can translocate the gut–blood barrier and cause bacteremia in patients with leukemia [9]. *Bacillus* spp. has also been reported as a cause of bacteremia in patients with hematologic malignancies and may be associated with increased mortality [10]. Furthermore, we observed cases of candidemia, which likely reflect the profound immunosuppressed state of our population—findings that are consistent with those reported in another retrospective study from Thailand [11]. In addition, although fungemia is suspected to contribute to mortality, we believe that its lack of statistical significance in the multivariate analysis may be due to the small sample size, which limits the ability to detect an independent effect.

In previous study had shown that the SOFA score exhibits the highest predictive validity for hospital mortality, with a particularly strong performance when the score is ≥ 6, showing a moderate positive likelihood ratio of 2.75 for hospital mortality [12]. Similarly, in our study, we identified that inadequate resuscitation leading to a post-resuscitation lactate level persistently exceeding 4 mmol/L is associated with increased mortality. Our study reaffirmed that SOFA score and lactate levels are independent predictors of outcomes in the management of bacteremia and sepsis, even after adjusting for other potential factors such as candidemia. This is particularly relevant among immunocompromised patients—an area that remains underexplored. Our findings highlight the importance of timely and adequate resuscitation in this population to improve clinical outcomes.

Additionally, our study has identified that underlying COPD or asthma serves as a protective factor. This is likely attributed to the admission of these patients with non-severe sepsis, but these patients are vulnerable to ICU admission due to exacerbation or attacks of their COPD or asthma resulting from the infection. Furthermore, ICU at our facility specializes in pulmonary care, contributing to better outcomes, which may help reduce mortality in this specific group of patients. This contrasts with a previous study that reported a notable increase in both ICU mortality (13.5% vs. 8.9%) and in-hospital mortality (17.1% vs. 12.3%) among patients with COPD. Additionally, patients with COPD in that study demonstrated higher mortality rates at 7-, 14-, and 21-days post-admission [13].

A major strength of our study is its focus on immunocompromised patients, who are often excluded from several studies despite their vulnerability to infections, particularly BSI. Our facility specializes in managing immunocompromised patients, providing us with a great opportunity to explore this unanswered question. Additionally, we explored several factors that impact mortality. Standardized criteria make this study valid and objective in terms of vital sign evaluation. However, limitations cannot be ignored. The retrospective nature of the design could not avoid recall bias; however, we attempted to adjust for this in the analysis with propensity score matching, which provides better comparability in demographic data. Additionally, evaluating appropriate antibiotic coverage is not thoroughly assessed and could significantly impact mortality. However, immunocompromised patients diagnosed with bacteremia at our hospital were consistently managed by infectious diseases specialists and were therefore expected to receive appropriate antimicrobial therapy.

In conclusion, our study highlights the prevalence of bloodstream infections, particularly stemming from gram-negative bacteria, among immunocompromised patients in the medical ICU at a tertiary care center. Major risk factors for mortality within 30 days include severe conditions characterized by higher SOFA scores and A lactate level above 4 mmol/L after 6 hours of resuscitation resulting from inadequate resuscitation. Notably, pulmonary underlying diseases emerge as a protective factor. Thus, further investigation and analysis of sepsis scores for predicting mortality are warranted.

## Supporting information

Supporting File 1 Concise patients’ data. (XLSX)
